# Bridging polarization generation and analysis on a photonic chip

**DOI:** 10.1038/s41377-026-02447-7

**Published:** 2026-08-26

**Authors:** Francesco Morichetti

**Affiliations:** Dipartimento di Elettronica, Informazione e Bioingegneria, Politecnico di Milano, 20133 Milano, Italy

**Keywords:** Integrated optics, Silicon photonics

## Abstract

Polarization is one of the most widely exploited degrees of freedom in photonics, yet controlling it within integrated circuits remains challenging. A reconfigurable silicon photonic circuit now combines polarization generation and analysis on a single chip, opening new opportunities for advanced communications, sensing, and optical information processing.

## Abstract (web summary)

Integrated silicon photonic polarization synthesizer/analyzer using a self-configuring MZI mesh, enabling arbitrary polarization generation, manipulation, and reconstruction

## Graphical abstract

**Figure:**
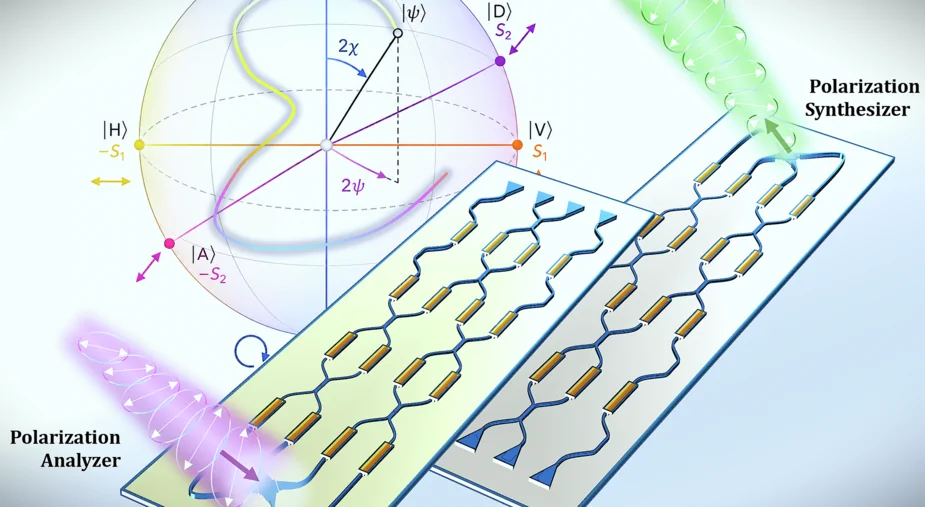

Polarization is a fundamental property of light. It can be exploited to carry information in optical communication systems, provide contrast mechanisms in imaging and sensing, and offer a key degree of freedom in quantum photonics. Despite its widespread use, the generation, manipulation, and analysis of the polarization states of the light continue to stimulate active research. One of the main challenges is that polarization control still relies largely on bulky optical components such as waveplates, polarizers, beam splitters, and free-space interferometers. As photonic systems become increasingly integrated and scalable, the challenge is no longer simply to generate or measure polarization states with high accuracy, but to do so within small size photonic chips. Such integration would enable polarization-based communication and sensing techniques in low-cost, portable, and even wearable devices.

Recent advances in photonic technologies have enabled remarkable progress toward the integration of increasingly complex functionalities on chips. Programmable photonic meshes of Mach–Zehnder interferometers (MZIs) can implement arbitrary linear optical transformations and realize universal programmable photonic circuits^1,2^. These architectures have proven highly effective for the generation^2^, analysis^3,4^, and sorting^5^ of structured light beams, the separation of orthogonal spatial modes^6^, the detection of wavefront-distorted beams^7^, and the identification of optimal communication channels in arbitrary optical systems^8^.

In their work^9^, Valdez and colleagues present a photonic integrated architecture capable of both generating and analyzing arbitrary polarization states within a single chip (see Fig. 1). The system combines a four-port polarization-splitting grating coupler with a self-configuring MZI mesh, creating a unified platform that provides programmable access to the full polarization degree of freedom. A key innovation lies in the use of a polarization-splitting grating coupler working at normal incidence, which enables optimal coupling conditions for both polarization states, thereby reducing the insertion loss and simplifying optical alignment with free-space beams.

**Fig. 1:**
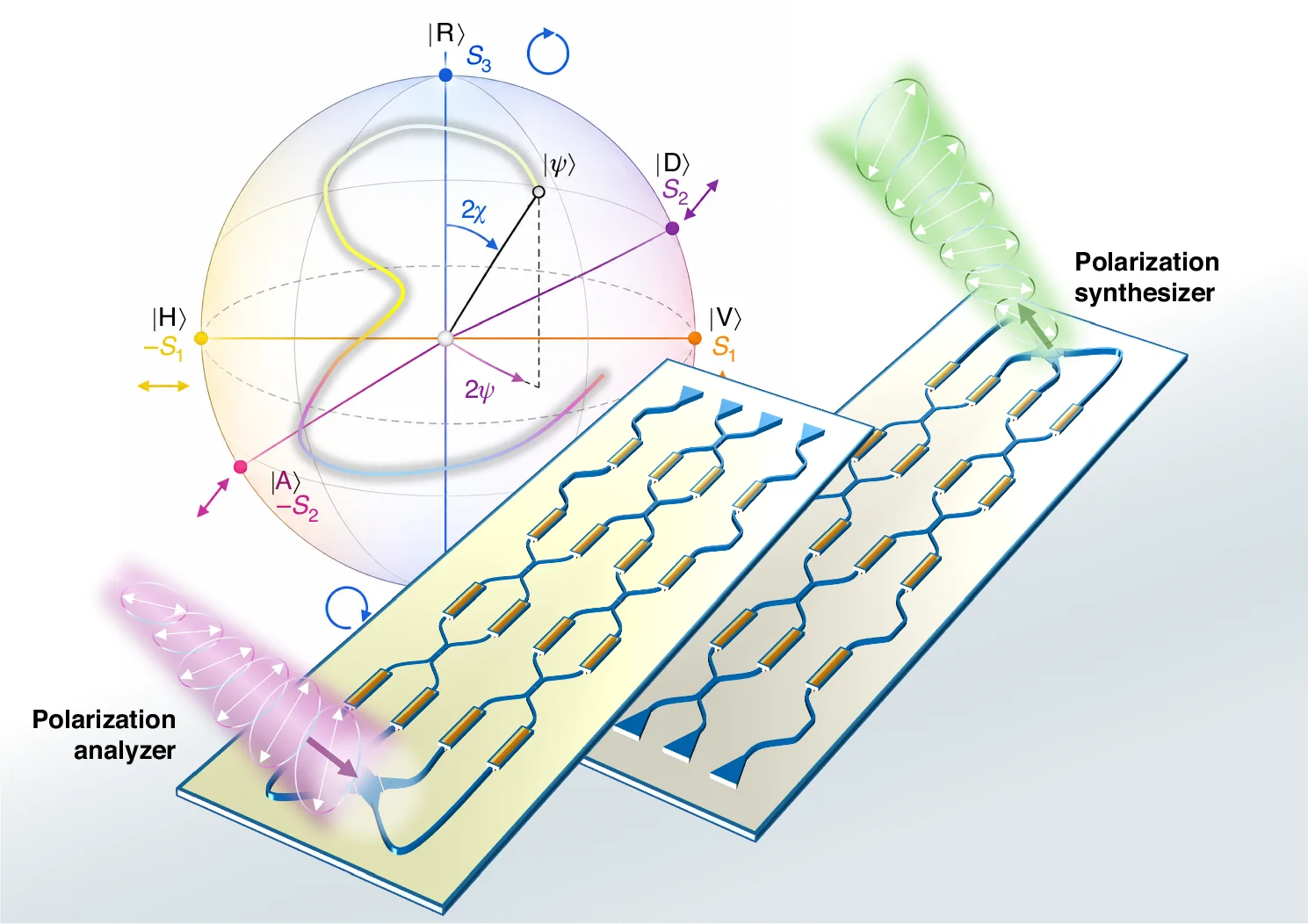
Integrated photonic polarization synthesizer and analyzer. The silicon photonic circuit integrates a four-port polarization-splitting grating coupler and a self-configuring binary-tree mesh of MZIs. This reconfigurable architecture provides full manipulation of the polarization degrees of freedom and can operate bidirectionally. In the forward direction, the interferometric mesh controls the relative amplitudes and phases of the optical fields to synthesize arbitrary polarization states. In the reverse direction, the same circuit analyzes an incoming polarization state by interferometrically recombining the polarization-demultiplexed signals, enabling reconstruction of the incident polarization without direct detection of the optical beam

When operated in the forward direction, the circuit synthesizes arbitrary polarization states by controlling the complex amplitudes delivered to the grating coupler. When operated in reverse, the same circuit analyzes the incoming polarization state through interferometric recombination, extracting the Stokes parameters directly from the calibrated phase settings of the photonic mesh. The accuracy of the polarization measurement is on the order of a few degrees on the Poincaré sphere, comparable with other integrated photonic implementations and approaching the performance of bulk optical systems, which typically provide sub-degree accuracy.

Unlike conventional polarimeters, this approach enables polarization analysis without directly detecting or absorbing the optical signal itself. Traditional polarization measurements rely on intensity detection after a sequence of polarization projections, inevitably consuming the optical signal. In contrast, the interferometric strategy demonstrated here preserves the optical field for subsequent processing. Such a capability could become highly attractive in spectroscopy, coherent communications, adaptive sensing, and quantum information systems.

This achievement is the result of a progressive evolution that we have observed in the integration of devices for polarization generation, control, and analysis. Previously reported devices perform either polarization analysis or polarization control, but not both within the same photonic platform. Table 1 summarizes and compares the most relevant contributions in this field. One of the first examples of an integrated polarization controller was proposed by Velha et al.^10^, who demonstrated a broadband silicon-photonic circuit capable of both generating arbitrary states of polarization and compensating incoming polarization states by recombining the components into the transverse-electric (TE) mode of an optical waveguide. Lin et al.^11^ demonstrated one of the first chip-scale full-Stokes polarimeters capable of reconstructing arbitrary polarization states directly on a silicon photonic platform. This device relies entirely on passive components and lacks the reconfigurability required for polarization generation or control. This work was subsequently extended to spectropolarimetric measurements by integrating thermally tuneable microring resonator filters on the same chip^12^, thereby enabling simultaneous spectral and polarization characterization. More recently, Stockinger et al.^13^ extended integrated polarimetry into the visible spectral range using a silicon nitride platform and proposed a robust calibration framework to compensate for fabrication imperfections, polarization crosstalk, phase errors, and non-ideal grating-coupler behavior. Within this landscape, the architecture reported by Valdez et al. is the first integrated circuit capable of both synthesizing arbitrary polarization states and analyzing unknown polarization states using the same hardware platform.

**Table 1:** Comparison of integrated photonic devices for the analysis and synthesis of arbitrary polarization states

Work	Device type	Analysis	Generation/control	Reconfigurability
Velha et al.^10^	Polarization Controller	No	Yes	Yes
Lin et al.^11^	Full-Stokes Polarimeter	Yes	No	No
Lin et al.^12^	Spectropolarimeter	Yes	No	No
Bütow et al.^4^	Amplitude/phase /polarization analyzer	Yes	No	Yes
Stockinger et al.^13^	Visible-light Polarimeter	Yes	No	No
Valdez et al.^9^	Polarization Synthesizer and Analyzer	Yes	Yes	Yes

However, the story is far from finished, and significant opportunities remain to further improve on-chip polarization management. The current implementation relies on thermo-optic phase shifters, which consume power (18 mW per actuator) and have a limited tuning speed ( ~ 30 μs) compared with electro-optic actuation systems. Electro-optic actuation, exploiting carrier-depletion silicon modulators^14^, thin-film lithium niobate^15^, or MEMS-based phase shifters^16^, could provide orders-of-magnitude improvements in switching speed and energy efficiency. Such improvements would be particularly beneficial for applications requiring rapid polarization tracking and low-power operation, including high-speed coherent optical communications, free-space optical links, adaptive optics, LiDAR, satellite communications, and polarization-multiplexed transceivers. Likewise, the demonstrated polarization-splitting grating coupler exhibits insertion losses of approximately 4.5 dB, which may be limiting for highly loss-sensitive applications such as quantum photonics and astro-photonics. Future implementations employing multi-etch gratings, dual-layer structures^17^, bottom reflectors^18^, or additional material layers^19^ could significantly improve coupling efficiency while simultaneously reducing reflections, scattering, and polarization reconstruction errors.

Looking ahead, photonic systems are expected to simultaneously harness more and more degrees of freedom of light, including wavelength channels^20^, spatial modes^2–6,8^ coherence^21^, and polarization^9^. The rapid progress in programmable integrated photonics is demonstrating that functionalities once implemented using complex assemblies of bulk optical components can now be realized on single reconfigurable chips^1^. In this context, the integrated polarization synthesizer and analyzer reported by Valdez and colleagues marks an important step toward multifunctional photonic processors with integrated polarization management.
